# Tracing embodied word production in persons with Parkinson’s disease in distinct motor conditions

**DOI:** 10.1038/s41598-022-21106-6

**Published:** 2022-10-05

**Authors:** Fabian Klostermann, Michelle Wyrobnik, Moritz Boll, Felicitas Ehlen, Hannes Ole Tiedt

**Affiliations:** 1 Department of Neurology, Motor and Cognition Group, Charité – Universitätsmedizin Berlin, corporate member of Freie Universität Berlin and Humboldt-Universität zu Berlin, Hindenburgdamm 30, 12200 Berlin, Germany; 2 Berlin School of Mind and Brain, Humboldt-Universität zu Berlin, Unter den Linden 6, 10099 Berlin, Germany; 3 Institute of Psychology, Humboldt-Universität zu Berlin, Rudower Chaussee 18, 12489 Berlin, Germany; 4 Department of Psychiatry, Jüdisches Krankenhaus Berlin, Heinz-Galinski-Straße 1, 13347 Berlin, Germany

**Keywords:** Cognitive neuroscience, Movement disorders

## Abstract

Embodied cognition theories posit direct interactions between sensorimotor and mental processing. Various clinical observations have been interpreted in this controversial framework, amongst others, low verb generation in word production tasks performed by persons with Parkinson’s disease (PD). If this were the consequence of reduced motor simulation of prevalent action semantics in this word class, reduced PD pathophysiology should result in increased verb production and a general shift of lexical contents towards particular movement-related meanings. 17 persons with PD and bilateral deep brain stimulation (DBS) of the subhtalamic nucleus (STN) and 17 healthy control persons engaged in a semantically unconstrained, phonemic verbal fluency task, the former in both DBS-off and DBS-on states. The analysis referred to the number of words produced, verb use, and the occurrence of different dimensions of movement-related semantics in the lexical output. Persons with PD produced fewer words than controls. In the DBS-off, but not in the DBS-on condition, the proportion of verbs within this reduced output was lower than in controls. Lowered verb production went in parallel with a semantic shift: in persons with PD in the DBS-off, but not the DBS-on condition, the relatedness of produced words to own body-movement was lower than in controls. In persons with PD, DBS induced-changes of the motor condition appear to go along with formal and semantic shifts in word production. The results are compatible with the idea of some impact of motor system states on lexical processing.

## Introduction

Motor Cognition (MC) is a controversially discussed theoretical paradigm, conceptualizing various mental functions as grounded in movement-related processing^1,2^. It contrasts with the idea that cognitive operations are combinatorial computations in an abstract sign code without any connection to sensorimotor processing^3,4^. As in other theories of embodied cognition (EC), in MC motor simulation is thought to be instrumental for the understanding of, e.g., gestures or semantic concepts related to movement^5–7^. Typical findings discussed in this general framework are, for instance, motor cortex activation upon perceiving action-related words^8^, resemblance of electroencephalographic activity during motor performance and observation^9^, or language processing facilitated by the simultaneous execution of ‘congruent’ movements^10^. In an ongoing debate, this principle concept has been impugned^2,11,12^. Criticized points are, amongst others, biased interpretation of weak effects of questionable significance, the assumption of motor-to-cognitive relatedness based on the co-occurrence of phenomena which are not necessarily interdependent, failed attempts to reproduce results central for the overall theory, and doubts about the appropriateness of MC and EC approaches to explain high-order cognition in complex real word scenarios^11,13,14^.

In view of pathological conditions, MC was predominantly discussed in the context of neurological movement disorders. In a reverse conclusion from the mentioned premises, motor system impairment was posited to hamper the processing of information with movement-related connotations^15–18^, and this was presumed to impact on various cognitive capacities up to abstract reasoning, depending on the particular formulation of the theory^19^. Clinically, MC positions are of particular interest in the context of Parkinson’s disease (PD), a prevalent condition with both motor and cognitive symptoms^20^. Although widespread cortical involvement in advanced disease stages may easily explain this association^21^, less overt cognitive symptoms already prevail in early PD with leading bradykinetic symptoms and nigrostriatal brain affection^22^. From the perspective of MC, this seems to support model claims, but, of course, other explanations are possible.

The ambiguity of interpreting subtle cognitive changes in PD can be illustrated using the example of particular biolinguistic findings^23^. It has been shown that persons with versus without PD differ from each other in view of verb processing across a variety of experimental conditions, implying naming, lexical decisions, comprehension and generation^18,23–26^. Under the premise of higher semantic association of verbs than other word classes with motor action, various researchers discussed this observation as an indication of MC. Alternatively it has been suggested that word production involves distinct neuroanatomical networks, depending on the lexical class processed. In view of verb generation, particular relevance has been ascribed to frontal opercular brain regions as a target of basal ganglia input. PD is thought to cause dysfunctional signaling in this network related to cognitive control functions^27,28^, e.g., lexical selection out of the numerous word forms of verbs^29,30^. Thus, altered use of lexical classes could reflect impaired striatal-frontal processing without any association to the motor system^28,31–33^.

To delineate in which ways PD impacts on word use, content-related lexical analyses on top of word class assessments may add valuable information. Following central MC claims, the ongoing grade of motor impairment in persons with PD should leave a ‘corresponding’ trace in mental concepts, for example, emerging as lexical output in semantically unconstrained verbal fluency tasks^34^. Of note, strongly contrasting movement conditions do occur in one and the same person with PD as a function of the actual therapy. The best match between clinically observable condition and motor system state is inherently connected with deep brain stimulation (DBS), comprising a neuroanatomically well-defined mechanism of action. In case of PD, high frequency electrical impulses are mostly bihemispherically delivered to the motor zone of the subthalamic nucleus (STN), which is a core basal ganglia structure promoting pathological signaling in PD^35^. Activation or inactivation of DBS of the STN leads to increased or decreased motor function, respectively, by modulating motor network states either towards a physiological operating mode (DBS-on) or away from it (DBS-off)^36^. In doing so, the changes of the clinical movement disorder evolve more rapidly and, thus, are better observable than upon starting or stopping pharmacological PD treatment due to prolonged drug-washout^37^.

Coming from a neutral, yet interested position with respect to MC concepts, we strived to analyze whether words produced by persons with versus without PD differed from each other with respect to their semantic movement-relatedness and whether this depended on the motor system state the persons with PD were momentarily in (i.e., DBS-on versus DBS-off). Thereby, we focused on lexical associations with (i) movement in general, (ii) own-body-movement, (iii) movement of objects, and (iv) movement of other beings, under the idea that a potential influence of the motor state on these dimensions could inform about issues outlined above. Besides, we tested whether the previously reported finding of lowered verb production in persons with PD would also be suggested by the current data and if such a potential finding was modified by the DBS and, thus, motor condition. In a straightforward understanding of MC, we would expect that motor system states reducing one’s own (not others') mobility should reduce lexical own-body movement-relatedness. In an extended MC view, seeing motor simulation as involved in the processing of any movement-related semantics, hypokinetic states should additionally affect the relatedness of words to external movements. Finally, as a non-MC, amodal language network effect, decreased verb use should be associated with poor motor states (as formerly reported), but not with a change of lexical relatedness to a particular dimension of movement.

To differentiate between these possibilities, word output was assessed in healthy persons and in persons with PD on versus off DBS of the STN, who engaged in a phonemic verbal fluency task without semantic constraints. Raters, blinded for the origin of the cumulated, random order lexical list, evaluated the relatedness of each word produced to the movement dimensions mentioned above. The group and state-related results of these ratings were critically weighed against positions pro and contra MC in view of potential associations with the behavioral condition and motor system state.

## Method

We analyzed data from 17 persons with PD and bilateral DBS of the STN and 17 age-matched healthy volunteers as controls. All participants gave written informed consent to the study protocol approved by the ethics committee of the Charité – University Medicine Berlin (protocol number EA2/047/10) and the study was performed in accordance with relevant guidelines and regulations in accordance with the Declaration of Helsinki. Persons with PD fulfilled the diagnostic Brain Bank Criteria and were treated in the Outpatient Clinic for Movement Disorders at the Department of Neurology of the Charité – Universitätsmedizin Berlin. Exclusion criteria for persons with PD were brain diseases other than PD including depressive or psychotic disorders (according to the diagnostic criteria of the German Manual for Psychopathological Diagnosis, AMD), or major cognitive decline as indicated by less than 15 points in the Parkinson Neuropsychometric Dementia Assessment (PANDA). The latter is a screening measure designed for the detection of cognitive impairments in persons with PD, which includes five cognitive subtests, i.e., (i) a word learning task, (ii) alternating phonemic VF, (iii) a visuospatial task, (iv) a working memory and attention task, and (v) delayed recall of the word list^38^. To control for the known and selective VF deficit in persons with PD and STN DBS, we also calculated the PANDA score except the VF subtask therein (the ‘net-PANDA’^39^) in addition to the total PANDA score to gain an orienting value of cognitive function apart from VF being the subject of this study. For comparability, all healthy controls also completed the PANDA; we did not include the mood questionnaire as the screening for study participation included a more detailed clinical assessment of potential psychopathology (see above). Volunteers in the control group were free of neurological or psychiatric pathologies. All participants were native German speakers. The participants with PD were treated with bilateral DBS of the STN and dopaminergic medication. For both groups, the relevant demographic information and PANDA scores as well as clinical data for the PD group are summarized in Table 1. To test for potential group differences in these parameters, we used independent samples *t*-tests or Wilcoxon-rank-sum-tests for non-dichotomous data (PANDA scores, age, years of education), depending on the data distribution as indicated by the Kolmogorov–Smirnov test. The chi-square test was used for dichotomous data such as sex ratio within each group or handedness.

**Table 1:** Demographic and clinical sample characteristics.

	PD group	Control group	Statistics
Age (years)	65.1 (9.1)	66.9 (8.1)	*p* = *.519*
Education (years)	10.0 (1.5)	10.7 (2.0)	*p* = *.256*
PANDA total (points)	20.4 (5.6)	24.9 (3.5)	*p* = .008
netPANDA net (points)	16.1 (4.6)	18.6 (3.2)	*p* = *.072*
Sex ratio (female/male)	4/13	4/13	*p* = *1.0*
Handedness (left/right)	2/15	3/14	*p* = *.628*
**Clinical characteristics of the PD group**			
Side of onset (left/right/bilateral)	3/13/1		
Disease duration (years)	15 (5.5)		
DBS duration (years)	3.6 (2.2)		
UPDRS III DBS-on	21.0 (9.5)		
UPDRS III DBS-off	39.5 (14.2)		
LEDD DBS (mg)	553 (399)		

Values are the mean with standard deviations in brackets. PANDA scores were raised in the DBS-on state of persons with PD. *PANDA* Parkinson neuropsychometric dementia assessment, *UPDRS* Unified Parkinson’s disease rating scale, *LEDD* Levodopa equivalent daily dosage.

### Electrode implantation and localization

DBS electrodes were implanted in the department of neurosurgery of the Charité – University Medicine Berlin (tetrapolar Lead Model 3389; Medtronic). Bilateral STN placement relied on atlas coordinates for the nucleus together with preoperative MRI, intraoperatively guided additionally by micro-electrode recordings and the effect of macro-electrode test stimulations. Localization was controlled by postoperative T2w-MRIs. For determining final electrode positions, the post-operative MRI-data was normalized to the Montreal Neurological Institute (MNI) stereotactic space, and the medio-lateral (x-axis), anterior–posterior (y-axis) and rostro-caudal (z-axis) distances of the susceptibility artifacts from the active electrode-contacts to the central MNI reference point were assessed. The specific values for localization and stimulation settings are provided in Table 2.

**Table 2:** DBS Parameters.

	Right STN	Left STN
Amplitude (V)	2.9 (1.3)	2.9 (1.2)
Pulse width (µs)	63.5 (9.9)	67.1 (13.1)
Frequency (Hz)	129 (33)	129 (33)
Impedance (Ω)	975 (543)	883 (463)
TEED_1sec_	107.2 (95.9)	122.9 (122.5)
Polarity (monopolar/bipolar)	13/4	15/2
Contact (single/double)	12/5	13/4
**Electrode localization**		
x-axis (mm)	11.64 (0.76)	− 11.82 (0.99)
y-axis (mm)	− 14.73 (1.04)	− 14.15 (1.05)
z-axis (mm)	− 7.13 (1.3)	− 7.23 (1.63)

TEED_1sec_ = total electrical energy delivered = (voltage^2^ × pulse width × frequency)/impedance.

### Task

We analyzed the lexical output of the participants in the non-alternating, phonemic version of the standard German Verbal Fluency (VF) task (Regensburger Wortflüssigkeitstest)^40^. Participants were asked to produce as many words as possible beginning with S during two minutes without any word class and semantic constraints. Word or word-stem repetitions, numbers and proper names were defined as errors, which was explained to the participants before the assessment started. The participants with PD performed the task twice, once with active DBS (i.e., DBS-on) and another time with the stimulation switched off for at least 30 min (i.e., DBS-off). These two assessments took place in two separate sessions at intervals of 8 weeks in counterbalanced order (DBS-on first versus DBS-off first), between which the medication remained unchanged. The motor conditions in the DBS-on and DBS-off condition were assessed by the Unified Parkinson Disease Rating Scale (UPDRS, part III)^41^. See Table 1 for the relevant clinical data including UPDRS III scores (DBS-on and DBS-off) as well as the levodopa equivalent daily dosage (LEDD) calculated according to Tomlinson et al.^42^.

The non-alternating phonemic VF task was part of a full assessment of VF abilities including an alternating phonemic VF task and (alternating and non-alternating) semantic VF as well. Words from VF conditions other than non-alternating phonemic VF were not subjected to the analysis reported here, since they were incompatible with the current study aim. For instance, in the semantic non-alternating subtask words had to denominate vegetables and in the semantic alternating subtask animals and pieces of furniture, so that word search was restricted to nouns of predefined semantic categories, not allowing for variations of lexical classes and movement-relatedness. In the alternating phonemic subtask (words beginning with G and R) executive processing demands were imposed next to lexical processing demands. However, we sought to avoid any cognitive complexity, which could have interfered with the operations in question as a potential confounder of the results.

As formal parameters of task performance in the non-alternating phonemic subtask, the mean number of words (i.e., total as well as correct words) and error-rates (i.e., percentage of false responses per produced words) were determined. The production of nouns and verbs was differentiated as percentage values with respect to total word counts to provide comparability between groups, since PD patients with DBS regularly produce less words than controls in VF tasks.

Further, an analysis of motor-relatedness of the generated words was performed. We analyzed the lexical output from all study participants including false responses, which inform about underlying semantic concepts as correct responses do^43^. Any word produced was listed once, regardless of whether it was named by different study participants, resulting in a corpus of 728 different words. Since there is no German word corpus on the lexical contents of interest here, this word list, inter-individually randomized in its order, was presented to 16 healthy native German speakers who had to evaluate defined semantic aspects^34^. These raters were not aware of the origin of the words or the aim of the study, and had to rate any single word under four motor-related semantic aspects on a 0–10 scale (minimum 0 = no association at all, maximum 10 = strongest possible association). The word dimensions to be rated addressed the MC-derived question whether particular motor system states, different between groups and DBS conditions, impacted on movement-associated word contents. Since the evaluation of lexical movement-relatedness in general was deemed as too unspecific, own-body and external perspectives were additionally accounted for. Thus, any listed word had to be rated with respect to its relatedness to (i) movement in general (e.g., comparably high for BIKE, comparably low for TOWER), (ii) movement of the own body (e.g., comparably high for FOOT or THROW, comparably low for AIRPLANE or THINK), (iii) movement of another living object (e.g., another human or animal, e.g., words like PET or FLY), and (iv) movement of an inanimate object (e.g., comparably high for BALL, comparably low for HOUSE). Similar examples using words that were not part of the corpus presented to the raters were given as part of the instructions beforehand, and the meaning of different movement dimensions was explained to the raters both in written and oral form. The resulting 2.912 evaluations (728 words in four dimensions) per rater were provided without a time limit, mostly demanding about six hours for completion, distributed over an individually defined number of sessions. Raters were instructed to mark words with unknown meaning, so that entries with less than eight ratings (< 50% of the available evaluations) could be excluded (see results section). For the remaining words, mean values of the ratings were calculated for further analysis^34^.

We report the inter-rater reliability for each movement-related dimension across the 16 raters according to Krippendorff’s Alpha^44^. Krippendorff’s Alpha is unaffected by the number of raters and can be applied to nominal, ordinal, interval and ratio data. Moreover, it allows for good reliability measures despite of missing data points in a fully-crossed design^45^.

### Statistical analysis

Potential differences of task performance (total and only correct word counts), task accuracy (error rates), lexical class use (percentage of verbs and nouns), and the above ratings of movement-related semantics were explored between the control group and the PD groups in the DBS-on and DBS-off conditions, respectively, and within the PD group, that is, between the DBS-on and DBS-off condition. Comparisons within the PD group and group-comparisons were conducted with paired-samples and independent-samples *t*-tests (we report *t*, degrees of freedom and *p*-values) and non-parametric tests, i.e., Wilcoxon signed-rank test and Wilcoxon sum-rank test (we report *U*, *z,* and *p*-values) for not-normally distributed data as indicated by the Kolmogorov–Smirnov test.

For the ratings of movement-related word associations, we conducted a logarithmic transformation of values to obtain normally distributed data allowing mixed repeated measures analysis of variance (ANOVA) instead of single non-parametric tests for each of the four dimensions of movement-relatedness. Normal distribution of the transformed data was then confirmed by the Kolmogorov–Smirnov test. The mixed ANOVA included the within-subjects factor *movement-related dimension* (4 levels: general, own body, other body, and objects) and between-subjects factor *group* (2 levels: PD group and controls). This ANOVA was conducted twice for the PD group in each DBS condition (DBS-on/DBS-off). Furthermore, effects of the DBS within the PD group were analyzed by entering variables into a repeated measures ANOVA with the factor *movement-related dimension* as above as well as *DBS treatment* (2 levels: DBS-on and DBS-off). Significant main effects or interactions involving *group* or *DBS treatment*, respectively, are reported. Where indicated, post-hoc tests between groups or DBS conditions within the PD group were conducted by using independent-samples or paired *t*-tests, respectively. In case of violations of the sphericity assumption, the Greenhouse–Geisser correction method was applied.

The significance threshold of all statistical tests was *p* < 0.05; in case of post-hoc testing for significant interactions in the ANOVA, *p*-values were corrected for multiple testing by use of the Bonferroni method. For all results, effect sizes are reported as Cohens *d* for *t*-tests (*d* = 0.2: small effect; *d* = 0.5: medium effect; *d* = 0.8: strong effect) and R^2^ for non-parametric tests (R^2^ = 0.02: small effect; R^2^ = 0.13: medium effect; R^2^ = 0.26: strong effect). For the ANOVA, partial eta squared (η^2^) was included as an effect size measure (η^2^ = 0.01: small effect; η^2^ = 0.06: medium effect; η^2^ = 0.14: large effect). All magnitudes of effect sizes are given according to Cohen^46^.

All analyses were conducted in SPSS (Statistical Package for the Social Sciences Software version 27, IBM, Walldorf, Germany) and R Studio (2019, Version 1.2.5033).

## Results

### Sample characteristics

The PD group did not differ from the control group with respect to age (*t*(32) = 0.563, *p* = 0.519, *d* = 0.224), years of education (*t*(32) = 1.155, *p* = 0.256, *d* = 0.396) or handedness (χ^2^ = 0.234, *p* = 0.628); the sex-distribution was identical in both groups. The mean total PANDA scores were significantly lower in the PD group on STN-DBS (*t*(32) = 2.825, *p* = 0.008, *d* = 0.969). The netPANDA, however, did not differ significantly between the groups (*t*(32) = 1.867, *p* = 0.072, *d* = 0.639). As expected, individuals with PD showed marked improvement of motor symptoms by DBS, evidenced by the mean UPDRS III scores in the DBS-on compared with the DBS-off condition (*t*(16) = − 7.654, *p* < 0.001, *d* = − 1.856). An overview of the sample characteristics is given in Table 1.

### VF task performance

In view of VF task performance, the PD group produced significantly fewer words than the control group, both in comparison to the DBS-on (*t*(32) = 2.310, *p* = 0.027, *d* = 0.792) and the DBS-off (*t*(32) = 2.854, *p* = 0.008, *d* = 0.979) conditions (see Table 3). Note, that this pattern remained the same if only correct words were included in the analysis (DBS-on versus controls; *t*(32) = 2.620, *p* = 0.013, *d* = 0.899; DBS-off versus controls, *t*(32) = 2.914, *p* = 0.006, *d* = 0.999). There was no significant difference of the total word count between both DBS-on and DBS-off within the PD group (*t(*16) = 1.603, *p* = 0.129, *d* = 0.389); the result was similar for correct words (*t*(16) = 0.975, *p* = 0.344, *d* = 0.236). No significant differences were identified with respect to error rates, neither between groups (DBS-on versus controls: *U* = 178.500, *z* = − 1.198, *p* = 0.231, R^2^ = 0.04; DBS-off versus controls: *U* = 150.000, *z* = − 0.196, *p* = 0.844, R^2^ = 0.001), nor between treatment states within the PD group (*z* = − 1.563, *p* = 0.118, R^2^ = 0.14).

**Table 3:** VF task results.

	DBS-on	DBS-off	DBS-on vs DBS-off	Controls	DBS-on vs controls	DBS-off vs controls
Words total	17.9 (8.3)	15.9 (8.6)	*p* = *.129*	25.6 (11.0)	*p* = *.027*	*p* = *.008*
Words correct	15.7 (8.1)	14.6 (8.4)	*p* = *.344*	24.2 (10.7)	*p* = *.006*	*p* = *.013*
Error rate (%)	13% (13)	8% (12)	*p* = *.118*	6% (6)	*p* = *.245*	*p* = *.865*
Verbs (% of total word count)	11% (10)	5% (8)	*p* = *.035*	16% (15)	*p* = *.487*	*p* = *.029*
Nouns (% of total word count)	82% (14)	88% (11)	*p* = *.070*	71% (23)	*p* = *.184*	*p* = *.040*

Overview of the mean VF performance (standard deviation in brackets) in the phonemic, non-alternating task and associated *p* values for between and within group comparisons, respectively. Correct word count is computed as the number of total words minus false responses. Error rates were calculated as the percentage of false responses per total word count; verb and noun ratios were computed as the number of words belonging to the respective lexical class per total word count.

With respect to the lexical class of the individual VF output, by far more nouns than verbs were produced, regardless of the group, with small fractions of other word classes (i.e., adverbs, adjectives, false responses including names or numbers). Relative to all words produced, persons with PD in the DBS-off condition produced a significantly smaller proportion of verbs (*U* = 83.500, *z* = − 2.178, *p* = 0.029, R^2^ = 0.14) and more nouns (*U* = 204.000, *z* = − 2.058, *p* = 0.040, R^2^ = 0.12) than the control group. This group difference was not significant in the comparison between persons with PD in the DBS-on condition and the control group (percentage of verbs: *U* = 124.500, *z* = − 0.695, *p* = 0.487, R^2^ = 0.01; percentage of nouns: *U* = 183.000, z = − 1.330, *p* = 0.184, R^2^ = 0.05). The comparison between treatment states within the PD group showed reduced verb production in the DBS-off as compared with the DBS-on condition (*z* = − 2.103, *p* = 0.035, R^2^ = 0.26), whereas the difference regarding the production of nouns did not reach statistical significance (*z* = − 1.810, *p* = 0.070, R^2^ = 0.19). For an overview of these results, see Table 3.

### Analysis of motor-relatedness

**Figure 1:**
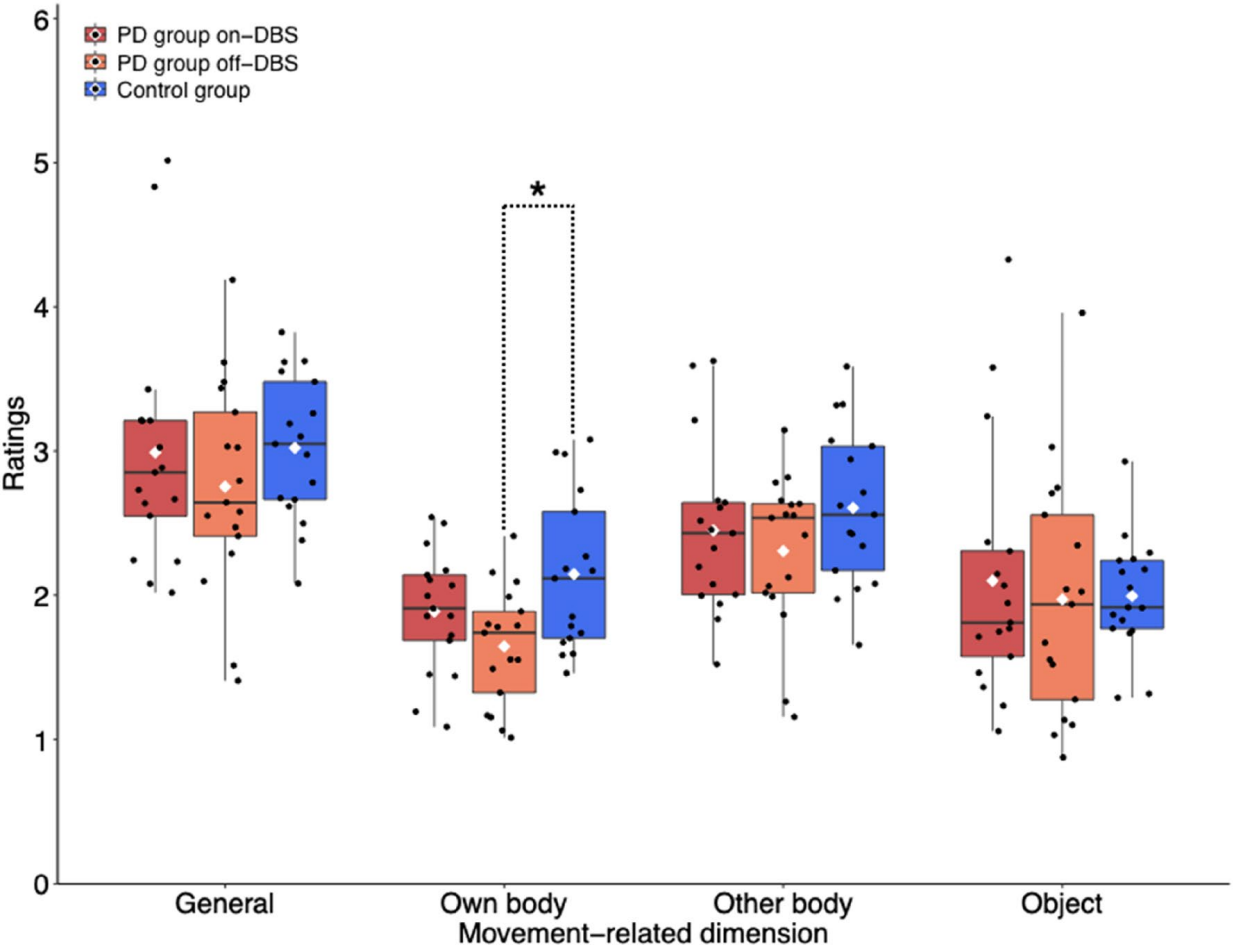
Ratings for the different dimensions of motor-relatedness of produced words per group and condition. Boxplots indicate the mean, upper and lower quartiles, and range. Dots display the individual means of the participants. The significant difference between groups is marked with an asterisk (*p* < .05). Note, that the full rating scale ranged from 0 to 10, however, the y-axis is abbreviated for a better display of the results, which only included mean ratings up to 5. The statistical analysis was conducted for the logarithmically transformed values.

From the 728 different words produced over all participants, 18 were marked as unfamiliar to the external raters, so that no semantic categorization was provided (2% of all words). The inter-rater reliabilities for the remaining 710 words across raters were low for the four movement related dimensions: general α = 0.356; own body α = 0.293; other body α = 0.315; objects α = 0.322. The mean ratings (before logarithmic transformation) per group as well as the distribution within each group can be taken from Table 4 and Fig. 1. The mean intra-rater reliability for the 16 raters was 0.614 (*SD* = 0.15).

**Table 4:** Mean ratings of word motor relatedness.

Movement relatedness dimensions	DBS-on	DBS-off	Controls
General	3.0 (0.8)	2.8 (0.7)	3.0 (0.5)
Own body	1.9 (0.4)	1.6 (0.4)	2.1 (0.5)
Other body	2.4 (0.6)	2.3 (0.5)	2.6 (0.5)
Objects	2.1 (0.9)	2.0 (0.8)	2.0 (0.4)

Values show the means and standard deviations (in brackets) of word motor relatedness per group and DBS condition.

The analysis of movement-related word associations yielded no significant main effect of or interaction involving the between-subjects factor *group* for the PD group in the DBS-on condition and controls. The ANOVA conducted for the PD group in the DBS-off condition and controls yielded a significant interaction of *group* and *movement-related dimension* (*F*(1.65, 52.8) = 4.046, *p* = 0.030, η^2^ = 0.112; df were Greenhouse–Geisser corrected). The main effect of *group* alone was not significant (*F*(1,32) = 1.619, *p* = 0.212, η^2^ = 0.048). Post-hoc comparisons of each of the four movement-related dimensions between both groups (DBS-off and controls) revealed that only the dimension of own-body related movement differed significantly (*t*(32) = − 2.796, *p* = 0.009, *p*_corrected_ = 0.036, *d* = 0.959) with lower ratings for the PD group (DBS-off) than for controls.

The ANOVA conducted within the PD group for effects of DBS on the motor relatedness of VF output yielded no significant main effect of *DBS* and no significant interaction of *DBS* and *movement-related dimension*.

## Discussion

In the current study, we investigated whether intra-individual motor system state changes influenced semantic and formal aspects of word generation. Therefore, persons with PD on as well as off DBS and healthy participants performed a phonemic VF task, so that lexical output could be analyzed per DBS condition and group with respect to different dimensions of movement-relatedness and word class properties.

In comparison to healthy participants, persons with PD in the DBS-off condition produced fewer words and, within this decreased lexical output, proportionally fewer verbs. Concerning this verb production difference, it should be noted, however, that it referred to very few words per participant, since the large majority of words produced were non-verbs in both groups and DBS conditions (see Table 3). Further, the words persons with PD generated in the DBS-off condition were rated as less associated with meanings implying own-body movement, whereas this was not true of other movement aspects. The group differences were no longer significant, when persons with PD were in the DBS-on condition. Between DBS conditions, no statistical distinction of VF-related performance was shown, while motor change, as assessed by the UPDRS, was significant. On average, values for own-body movement-relatedness and verb use in the DBS-on state were in between those measured in the DBS-off state in persons with PD and the corresponding values in healthy participants.

Principally, altered semantics and verb use could be conceived as a phenomenon without a specific relation to PD brain pathology, since everyday living conditions alone can frame lexical properties of persons or groups^47^. In PD, growing hypokinesia implies existential change related to agent-based motor behavior, and gradual alignment of mental concepts traceable on lexical levels could simply be a response to permanent mismatch between actual experience and unrealistic expectations^48–59^. However, if changed word use in persons with PD were only based on this, it should—as a slow, learning-based adaptation to enduring change—be inert to short-lived motor functional shifts by intermittent DBS in/activation. Yet, only in the DBS-off, but not in the DBS-on condition significant differences of lexical semantics and word class use were identified in comparison to persons without PD. Therefore, this framework does not comprehensively explain the obtained result pattern. Further, it is important to note that persons with PD experience the DBS-off condition as uncomfortable and, therefore, distress as a factor of the current results cannot be ruled out. However, this would probably have impaired VF task performance globally, i.e., as a reduction of word production without a relation to a specific semantic movement dimension or lexical class.

Concerning effects of acute motor change on lexical properties, two views deserve particular mention. Firstly, in the context of DBS modulation of word class use was conceptualized in a non-MC account, viewing low verb generation as a dysexecutive symptom in PD. Based on the assumption that striatal processing is crucial for inhibitory operations in motor as well as cognitive behaviors, PD was proposed to impair the release of words with numerous grammatical alternatives, as is the case in verbs. In this view, co-activation of their variable conjugational forms at the beginning of the lexemic retrieval process, demand the inhibition of all lexical candidates apart from the best-suited option. As this inhibitory selection process was presumed to be a frontostriatal function, its impairment could underlie verb production problems in persons with PD^27,29–31^. In this sense, the partial normalization of striatal function by DBS could have effectuated that the difference of verb production between controls and persons with PD in the DBS-off condition became insignificant under active DBS. This seems also compatible with previous findings, demonstrating that DBS of the STN not only acts on motor processing, but also supports impaired language-related executive operations, such as conceptual switching during word production^60–62^. However, this concept does not include the particular semantic result pattern.

In this regard, a second view deserves a mention, in which both altered verb-generation and content-related findings are the consequence of a DBS-induced shift of mental concepts related to the acute functional gain of the motor system and, thus, the capacity to move. Note that the specific production differences between persons with PD in the DBS-off condition referred formally to the lexical class of verbs and semantically to own-body movement-relatedness. The latter finding cannot be easily explained as the result of reduced production of verbs as a grammatical class^18,23–26^, because in this case equivalent effects on the other dimensions of movement-relatedness should have occurred. However, reminiscent of typical MC claims, the group difference of word ratings was only significant for the DBS-off condition and diminished in the DBS-on state, suggesting partial normalization of the underlying processes. Thus, it rather reflected the specific ‘semantic impact’ of the motor system state change, particularly related to the ability to move one’s own body. Further, it should also be pointed out that word production took place in a physically inactive state, that is, while seating on a chair, so that the described semantic effect was not dependent on a particular use of the motor system. This is compatible with the idea that the modulation of motor physiology in itself influences mental concepts associated with active movement^2,11,13,14^.

Data potentially supporting MC positions are controversially discussed. For example, the activation of motor cortical areas during action word processing in functional imaging^8,63^ may indicate ‘modal’ cognitive processing or, alternatively, a post-lexical phenomenon without functional relevance, the Action Sentence Compatibility Effect (ACE)^2,10^ is highly cited and, at the same time, principally put into question for a lack of reproducibility^13^ , and so forth. Against this background, the current data are of interest, because DBS of the STN brings the motor network of persons with PD in a closer-to-normal physiological state, observable as a rapidly evolving relief of clinical symptoms^64–67^. As known from many other investigations, it approximately halved the motor UPDRS in persons with PD, so that—according to this scale—the average movement function in the DBS-on condition was quite in the middle between the DBS-off state and the motor condition of persons without PD. Of note, lexical differences became only significant contrasting the UPDRS-defined motor conditions 40 points away from each other, but not at the also significant 20-point-distances, prevailing between persons with PD in the DBS-on state and persons without PD as well as within persons with PD on versus off DBS. Thus, whereas motor states may acutely affect the processing of words, this effect seems altogether relatively subtle.

**Figure 2:**
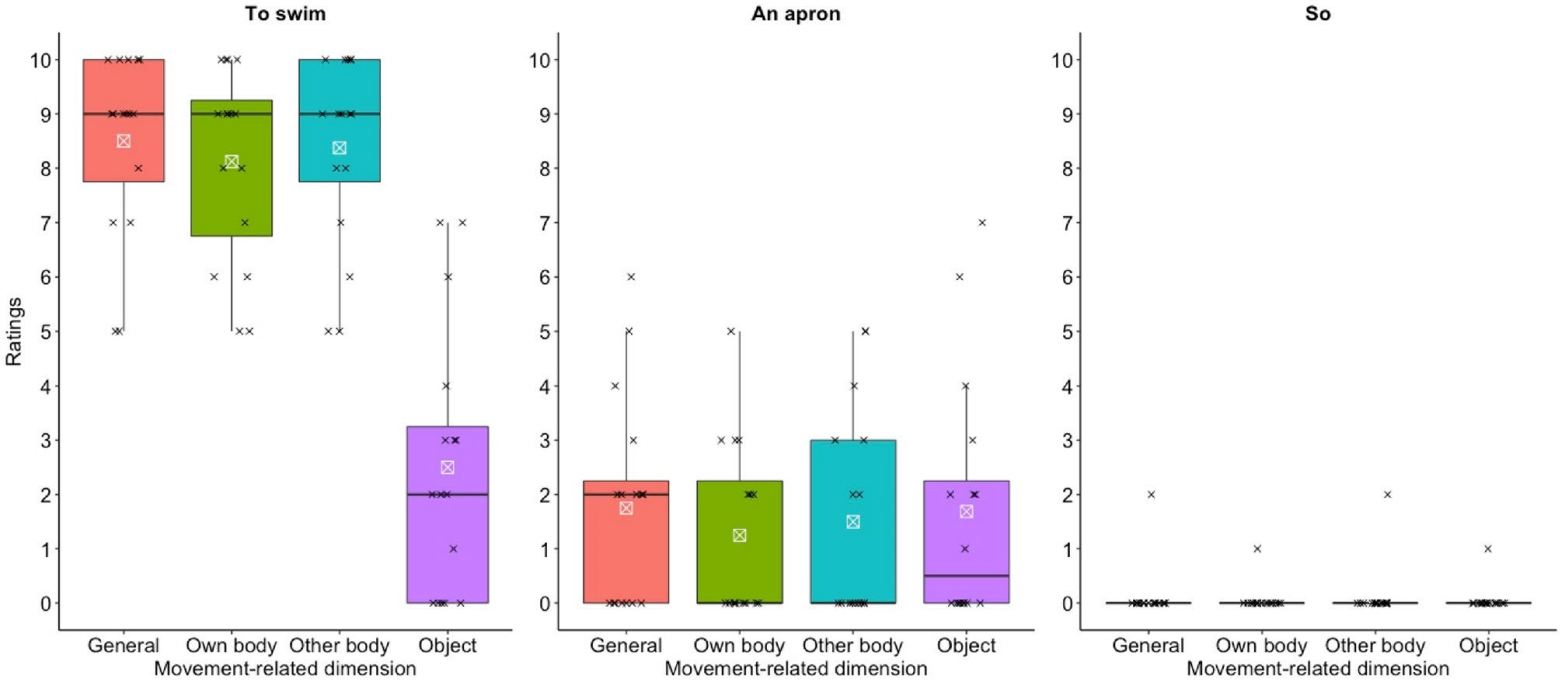
Individual ratings of the middle words from the lists (Table 5) for ‘highest’, ‘median’, and ‘lowest’ average ratings of own-body movement-relatedness. Cross signs indicate the per-rater scores.

Given the study limitations, these considerations are formulated with all the necessary caution. First of all, there is no German word corpus on lexical movement-relatedness, so that the semantics in question had to be derived from the ratings of a group of native German speakers, unaware of the data origin and the study aim^34^. On the used scale from 0 to 10 average ratings ranged around 2, and the observed group-difference of own-body movement-relatedness between persons with PD in the DBS-off state and controls was 0.5. Whether this small effect as well as the tiny differences in verb production has practically important implications remains unclear. In this regard, future studies using spontaneous speech samples could analyze potential effects of DBS on semantic levels of natural language. At this point, the current finding, raised in the artificial context of a VF task, may be considered as a signal of conceptual interest in the outlined theoretical context. Eventually, it should also be noted that, even in the logic of MC, it seems difficult to formulate an estimate of the degree of lexical-semantic change as a function of the severity of PD, in which motor system change is not absolute, but occurs gradually coming from a physiological basis of movement processing. Further, it needs to be mentioned that a very low inter-rater reliability was obtained, although the different dimensions of movement-relatedness and the used scale were extensively explained and corresponding examples were given. Principally, this could indicate that the rating task was difficult to accomplish or instructions were poorly understood. However, given the specific pattern of data material and ratings, we consider as likely that different persons naturally make quite divergent motor associations with words that were produced without any movement-related instruction and therefore convey rather low motor semantics. Accordingly, individual scores of motor-relatedness were mostly low, so that even minor differences of lexical conceptualization, e.g., due to inter-individually variable lexical use or experience associated with words, may have strongly decreased the agreement between the ratings. This thought is illustrated in Table 5, showing five words with the highest and lowest ratings of own-body movement relatedness each, as well as five words given the corresponding median rating value. The categorization of words with maximum and minimum values is easy to follow, just as the position of words with median ratings in between these extremes. However, the latter words (on the altogether low rating median) leave a wide interpretation space with respect to motor associations, probably implying factors such as personal habits, skills, attitudes, etc. In this regard, Fig. 2 shows the individual rating results for three example words, representative of the scoring pattern in case of high, medium, and low average scores for own-body movement-relatedness. With respect to the median score example (‘apron’), either half of the raters did or did not see movement-relatedness in any of the categories (an apron might move when one puts it on, but can be considered as immobile as well, and so on). This heterogeneity might reflect different associations the raters had with words, which do not primarily imply movement semantics, but which are interpretable in this regard, with average ratings of movement-relatedness between very high and low scores. The stronger homogeneity of ratings for words with maximum and minimum scores probably indicates less ambiguity with respect to the presence or absence of motor-related meanings. In so doing, the intra-rater reliability was moderate, indicating that, on an individual level, the evaluation pattern across the different dimensions of movement-relatedness varied between words to a certain degree, but not completely, i.e., the connection of one dimension of movement-relatedness to the other dimensions was not arbitrary per rater. Altogether, the raters judged words produced in the DBS-off state as less own-body movement-related than words produced by controls, but the evaluation of the movement-relatedness of each particular word strongly varied between them. To support the idea that this result pattern reflects a systematic embodiment effect, tasks used in future experiments should aim at higher inter-rater agreements, possibly directly demanding the production of movement-related words.

**Table 5:** Words with highest, median or lowest ratings.

	German	English translation	Mean rating
**The five highest rated words**			
1	Sport	Sports	8.438
2	springen	To jump	8.313
3	schwimmen	To swim	8.125
4	spurten	To sprint	7.643
5	Sprint	A sprint	6.813
**Five words with the median rating**			
369	Sack	A sack (i.e., a bag)	1.25
370	Steuer*	Tax or a steering wheel	1.25
371	Schürze	An apron	1.25
372	Schutz	Protection	1.25
374	Sitzmöbel	Seating furniture	1.25
**The five lowest rated words**			
719	Sorte	Variety; type	0.063
720	Superlativ	Superlative	0.063
721	So	So	0.063
722	Somit	Thus; therefore	0.063
723	Sie	She; they	0.063

Generated words with the five highest and lowest mean ratings and five words with the median rating in the own body movement dimension. *This German word has two meanings.

In sum, the results (abnormally low verb production together with decreased own-body movement-relatedness in persons with PD in the DBS-off condition; reduction of these differences to controls in the DBS-on condition) are compatible with the view that momentary motor system states impact on the availability or prevalence of corresponding mental concepts, traceable on the level of word output. In this formulation, interactions between motor and lexical processing could further be understood as a factor of low verb generation in PD, in addition and complementary to proposed dysexecutive underpinnings. The findings may serve as food for thought in the debate about the existence and nature of MC, but, of course, should be verified in further trials. With respect to the particular case of PD, they inspire to give some thought about the question whether certain cognitive sequelae of the condition might be less separable from motor dysfunction than commonly assumed.

## Data Availability

The datasets used and analysed during the current study are available from the corresponding author on reasonable request.
